# Use of a generic Paediatric Patient Reported Outcome Measure (P-PROM) in Routine hospital Outpatient Care for Kids (ROCK): A qualitative exploration of adolescent, caregiver and service provider perspectives (P-PROM ROCK Phase 1)

**DOI:** 10.1007/s11136-025-03990-3

**Published:** 2025-05-14

**Authors:** Renee Jones, Kim Dalziel, Harriet Hiscock, Alexander van Heudsen, Nancy Devlin

**Affiliations:** 1 Melbourne Health Economics, University of Melbourne, Victoria, Australia; 2 Health Services and Economics, Murdoch Children’s Research Institute, Victoria, Australia; 3 Department of Paediatrics, The University of Melbourne, Victoria, Australia

**Keywords:** Patient reported outcome measures, PROMs, Pediatrics, Clinical practice, Quality of care, Quality of life, EQ-5D-Y-5L, Qualitative, Implementation

## Abstract

**Purpose:**

To explore patient, caregiver, and service provider perspectives on the implementation of a generic Paediatric Patient Reported Outcome Measure (P-PROM), their perspectives on the EQ-5D-Y-5L, and to consider how these insights can be used to co-design routine use of EQ-5D-Y-5L in paediatric outpatient care.

**Methods:**

Individual semi-structured interviews were conducted with adolescent patients, caregivers, and providers (allied health, nurses, doctors) who had provided or received outpatient care at The Royal Children’s Hospital, Australia. Interview transcripts were analysed using framework analysis and subsequent themes were mapped to an acceptability framework.

**Results:**

Interviews were conducted in June and July 2023 with six service providers, five caregivers and three adolescent patients. Despite service provider concerns that generic P-PROMs may be too broad; all were open to their use. Participants did not see value in patients simply completing a generic P-PROM; it was emphasised that such information needed to be used and responded to. Participants were largely supportive of using the EQ-5D-Y-5L in clinical care, although some were concerned about the short recall period and negative framing. Displaying EQ-5D-Y-5L responses by item was preferred by participants. These different perspectives were summarised into enablers and barriers of acceptability.

**Conclusions:**

Perspectives gathered from this study highlight that generic P-PROMs, such as EQ-5D-Y-5L, may have potential for use in paediatric outpatient care. However, to have a meaningful impact, careful consideration is needed regarding implementation. Results have informed co-design of the P-PROM ROCK Program.

**Supplementary Information:**

The online version contains supplementary material available at 10.1007/s11136-025-03990-3.

## Plain English summary

There are some simple questionnaires that can be used to ask children (or their parents) about their overall health and how they are feeling. We think that this information could improve the care they receive from their doctors, nurses or other health care staff. The point of this study is to understand the views of children, their parents, and their doctors and nurses about this. We also wanted to get their views on a specific overall child health questionnaire that has five questions, known as the EQ-5D-Y-5L. This study found that children, their parents, and their doctors and nurses think that asking families about a child’s overall health using a questionnaire might help to improve their care. For example, they thought it could improve the communication between patients and their clinician, helping patients and their doctors to make better choices about treatments. However, this study also found that for the questionnaire to meaningfully improve a child’s care, it is important that families are given support to complete the questionnaire before their appointment, and that doctors and nurses are taught how to use this questionnaire information.

## Introduction

In recent decades there has been a shift towards patient-centred care, an approach that brings the patient and their voice to the centre of the health system [1, 2]. One way for health systems to engage in patient-centred care, is to understand patients’ needs by asking patients about their health, from their perspective, using tools such as Patient Reported Outcome Measures (PROMs). PROMs are standardised measures that capture a patient’s perspective of their health, functional status, wellbeing, or quality of life [3, 4]. When used in the patient-clinician encounter, PROMs have the potential to improve patient-clinician communication, patient satisfaction, patient engagement, health management decision making, and patient outcomes [5, 6].

PROMs can be either generic or condition-specific [3, 5, 7]. Generic PROMs capture aspects of health that are common to most populations [7]. Condition-specific PROMs capture aspects of health that are specific to a particular condition [7, 8]. Whilst both can be used in patient care, generic PROMs have the added benefit of being applicable across different health conditions and settings [5]. This can streamline implementation efforts and enable aggregation and comparison of PROM data [5, 9]. It is important to distinguish between the collection of generic PROMs and their use in clinical care. For example, generic PROMs may be collected to monitor health service performance or to evaluate the impact of an intervention at an aggregate level. Collecting generic PROM data from a patient is, in itself, unlikely to meaningfully impact the clinical care for that patient, unless there is careful implementation and use in clinical encounters [10]. The collection and use of generic PROMs in adult patient care is becoming increasingly common. However, the collection and use of paediatric specific PROMs (P-PROMs) in paediatric clinical care remains novel.

P-PROMs can be completed by either the child (self-report) or the child’s caregiver, usually a parent (proxy report). The gold standard is to obtain the child’s self-report where possible, however, this depends on the age and abilities of the child [11]. Few studies have assessed the use of P-PROMs in paediatric clinical care, with a 2020 systematic review identifying only seven studies [12]. Of these, three trialled a generic P-PROM and four trialled both a generic and condition specific P-PROM [12]. Where a generic P-PROM was trialled, this was mostly the 23-item PedsQL [12]. No studies included the EQ-5D-Y-5L or its predecessor, the EQ-5D-Y-3L. A key advantage of the EQ-5D-Y-5L in the clinical context is its brevity (only five items), making it quick to complete and minimising responder burden. The Australian Paediatric Multi-Instrument Comparison (P-MIC) study identified the median completion time of the EQ-5D-Y-5L in a sample of hospital participants to be 37 seconds [13]. The P-MIC study also highlighted the EQ-5D-Y-5L as one of the generic P-PROMs with the strongest psychometric performance [14]. Importantly, the EQ-5D-Y-5L was found to demonstrate responsiveness to both improvements and worsening health, whilst the PedsQL was found to only demonstrated responsiveness to worsening health [14]. When used in clinical care, it is important a PROM can pick up on changes in a patient’s health, including improvements and worsening health. Hence, evidence suggests that the EQ-5D-Y-5L may be an appropriate choice for inclusion in routine clinical paediatric care. However, given the EQ-5D-Y-5L and its predecessor, the EQ-5D-Y-3L, were largely designed for use in clinical trials to inform economic evaluations [15], further research is required to determine if the EQ-5D-Y-5L is appropriate for use in routine clinical paediatric care.

Prior to newly implementing a generic P-PROM in routine clinical care, it is essential to engage with local stakeholders to understand their perspectives on potential barriers and enablers to P-PROM acceptability [16, 17]. Barriers and enablers to the implementation of P-PROMs in paediatric care and health systems have recently been explored in two studies [18, 19]. A systematic review by Scott et al. (2023) report barriers including lack of staff knowledge about the impact of using P-PROMs in clinical care, challenges collecting, interpreting, and using P-PROM information in clinical care, and lack of resources, including both funding and staff resources, to support the sustainable implementation of P-PROMs in clinical care [18]. Frequently reported enablers included staff and family education about how to collect and use P-PROM information in clinical care, and the benefits of using P-PROMs compared to usual care [18]. A qualitative descriptive study by McCabe et al. (2023) identified key factors that affected implementation of P-PROMs and Paediatric Patient Reported Experience Measures (P-PREMs), including characteristics of P-PROMs and P-PREMs; individual’s beliefs; administering P-PROMs and P-PREMs; designing clinical workflows; and patient and clinician incentives [19]. The acceptability of the adult version of the EQ-5D instrument, the EQ-5D-5L, was qualitatively explored with Australian healthcare staff in a recent study, and identified that it was likely acceptable for routine use, as it was quick and easy to complete, measured multiple relevant aspects of health, identified patient problems, and measured service performance [20]. However, staff also identified some less acceptable features of the EQ-5D-5L in routine clinical care, including its highly generic items and short recall period [20]. Further research is needed that engages and understands the perspectives of parent and child consumers, as well as service providers, on the potential barriers and enablers to acceptability of using a generic P-PROM, such as the EQ-5D-Y-5L, in the paediatric clinical care context.

This study aims to explore adolescent patient, caregiver, and service provider (1) perspectives on the implementation of a generic P-PROM in clinical outpatient care, and (2) perspectives on the EQ-5D-Y-5L as a generic P-PROM for use in clinical care. This study also aims to understand how these perspectives can be used to co-design routine use of EQ-5D-Y-5L in clinical care. This study is Phase 1 of a wider project that aims to co-design, pilot and evaluate use of the EQ-5D-Y-5L in routine outpatient clinical care.

## Methods

### Study design

Qualitative methodology, involving semi-structured interviews, was used. Study methods are reported in accordance with the Consolidated Criteria for Reporting Qualitative Research (COREQ) [21]. This study is Phase 1 of a larger co-design project and learnings from this phase will inform the scope of subsequent co-design phases. Methods for this Phase 1 study and the latter Phase 2 study were informed by the co-design framework for public service design which includes the following seven steps: (1) resourcing, (2) planning, (3) recruiting, (4) sensitising, (5) facilitating, (6) reflecting, and (7) building for change [22]. Steps 1–3 (resourcing, planning, and recruiting) informed methods for this Phase 1 study, whilst latter steps informed methods for the Phase 2 study. Further details about these methods are described in the companion Phase 2 manuscript.

### Research questions

This study aims to explore the following Research Questions (RQs):

1. What are adolescent patient, caregiver, and service provider perspectives on the implementation of a generic P-PROM in clinical outpatient care?
2. What are adolescent patient, caregiver, and service provider perspectives on the EQ-5D-Y-5L as a generic P-PROM for use in clinical care?
3. How can these perspectives inform the co-design of routine use of EQ-5D-Y-5L in clinical care?

### Setting

The Royal Children’s Hospital is a publicly funded tertiary paediatric hospital in Melbourne, Australia. Outpatient care was the focus of this study as children attending these clinics are often chronically unwell, which is where PROM interventions are considered appropriate [23]. In Australia, outpatient care is provided via office-based visits at the tertiary paediatric hospital by specialised paediatricians.

### Participants

Participants were eligible if they were: (1) adolescents aged 12–18 years who were receiving or had received outpatient care in the previous 18 months, (2) caregivers of children aged 2–18 years who were receiving or had received outpatient care in the previous 18 months, or (3) service providers, including clinicians, nurses, or allied health staff, who were working in outpatient care.

Participants were recruited between May and July 2023. Service providers were recruited via email invitation using the study team’s network. Snowball sampling was also used, whereby service providers were asked to pass the research advertisement onto their colleagues. Adolescents and caregivers were recruited by emailing eligible caregiver participants from the Australian P-MIC study who had consented to hear about future research [24]. To enable recruitment of adolescents, caregivers of adolescents were asked if their adolescent would like to take part.

Participants were asked to complete eligibility questions and register their interest. Participants were purposively selected from those registering their interest [25], whereby participants who had more experience with paediatric outpatient care or from areas where future implementation of the P-PROM was planned (including colorectal surgery, asthma, sleep, and encopresis clinics) were prioritised.

### Data collection

Participants were asked to complete a brief online demographic survey prior to their interview.

Individual 30-minute online semi-structured interviews were conducted via video conferencing in June and July of 2023. Interviews were conducted by RJ, a female PhD student at The University of Melbourne. RJ is experienced in qualitative research, has a Master of Public Health, is not a clinician, and was known to two of the service provider participants. An interview guide was used to provide consistency in the way P-PROMs were introduced and to ensure key topics were covered (see Supplementary Material). Key topics included previous experience with P-PROMs in a health setting, views on generic P-PROMs in routine clinical care, views on the EQ-5D-Y-5L for use in routine clinical care, as well as challenges and opportunities. Adolescent participants were invited to have a parent or caregiver with them. Interviews were recorded, transcribed by an external company, de-identified, and checked for precision.

During the interview, all participants were shown the EQ-5D-Y-5L. The EQ-5D-Y-5L is a generic P-PROM that measures overall Health-Related Quality of Life (HRQoL). It comprises of five domains (mobility, self-care, usual activities, pain/discomfort, and feeling worried, sad or unhappy) and a Visual Analogue Scale (EQ VAS), asking about a child’s overall health on a scale of 0 to 100 [26]. The EQ-5D-Y-5L was developed to extend the number of response levels of the EQ-5D-Y-3L [26], which is validated for use in children aged 4–18 years [27]. The EQ-5D-Y-5L can be scored in a range of ways, including a simple level sum score approach,[28] and preference weighted scoring approaches [29, 30]. Different scoring approaches were discussed with participants during interviews. The EQ-5D-Y-5L was a focus of this study for several reasons: (1) funding was available to support the investigation of its use in clinical care, (2) it is short and quick to complete [13], (3) it has stronger measurement properties than the EQ-5D-Y-3L and other generic P-PROMs [14], and (4) it was adapted from the EQ-5D-5L, which has been used in routine clinical care in adults [29, 31, 32].

### Data analysis

Interview data were analysed using qualitative framework analysis, entailing seven stages: (1) transcription, (2) familiarisation with transcript, (3) coding, (4) development of framework, (5) application of framework, (6) charting into framework, and (7) interpreting data [33]. Interview data were coded using NVivo 12.0. To identify key themes, interview data were both deductively coded, using the interview guide (Supplementary File 1), and inductively coded, with additional categories coded as they appeared in the data. The interview guide was structured based on study research questions, and hence these research questions formed the backbone of the coding framework (see Supplementary File 1 for interview guide). Interviews were coded by both RJ and AvH. AvH is a male PhD student at the University of Melbourne, has a Master of Public Health, is trained in qualitative research, is not a clinician, and is not known to any participants. Coding was completed in rounds, whereby RJ and AvH would each separately double code several transcripts and then meet to discuss the analytical framework. This analytical framework was iteratively adapted as the rounds progressed. After three rounds, which involved the double coding of nine interviews by both RJ and AvH, the study team agreed on a final analytical coding framework that was applied to all interviews by RJ. RJ and AvH also discussed data and thematic saturation in each round, discussing the number of new codes and themes appearing [34]. By round three, no new codes had emerged, and no further interviews were conducted. Illustrative quotes were used to describe each theme. All quotes were pseudonymised and if required, truncated.

No formal sub-group analyses were conducted. However, if themes were determined by the study team to only relate to a certain participant group (i.e., if all quotes coded to a certain theme belonged to only one participant group), this was described in the results.

Themes identified from the interview data were mapped to the Theoretical Framework of Acceptability (TFA) conceptual framework after coding [35], similar to Snowdon et al. 2023 [20]. The mapping of themes to the TFA conceptual framework was done to investigate and summarise how the identified themes relate to the acceptability of using a generic P-PROM, specifically the EQ-5D-Y-5L, in routine clinical care. Clarifying the barriers and enablers of acceptability will inform latter co-design stages, ensuring key barriers are addressed and key enablers are maximised.

## Results

### Participant characteristics

Seventeen potential participants confirmed eligibility and registered their interest. Fourteen were selected, using purposive sampling methods described above, and completed an interview. Participant characteristics are described in Table 1.

**Table 1:** Participant characteristics

Participant characteristic	N (%) or mean (sd)
All participants, n = 14	
Completed interview, n(%)	14 (100)
Type of participant, n(%)	
Service provider	6 (42.9)
Caregiver of patient	5 (35.7)
Adolescent patient	3 (21.4)
Service provider participant characteristics, n = 6	
Type of clinician, n(%)	
Nurse	3 (50)
Doctor	3 (50)
Gender, n(%)	
Female	5 (83.3)
Male	1 (16.7)
Length of time working in outpatient clinic, n(%)	
3–5 years	1 (16.7)
5–10 years	2 (33.3)
> 10 years	3 (50)
Caregiver participant characteristics, n = 5	
Caregiver gender, n(%)	
Female	5 (100)
Child Gender, n(%)	
Female	1 (20)
Male	4 (80)
Child age in years, mean (sd)	7.6 (3.2)
Frequency of child’s appointments at outpatient clinic, n(%)	
Once off	1 (20)
Every 1–3 months	0
Every 3–6 months	2 (40)
> every 12 months	2 (40)
Adolescent participant characteristics, n = 3	
Adolescent Gender, n(%)	
Female	2 (66.7)
Male	1 (33.3)
Adolescent age in years, mean (sd)	15.3 (2.1)
Frequency of appointments at outpatient clinic, n(%)	
Once off	0
Every 1–3 months	2 (66.7)
Every 3–6 months	0
> every 12 months	1 (33.3)

### Key themes

Key themes for each research question (RQ), including illustrative quotes, are presented below.

- RQ1, What are adolescent patient, caregiver, and service provider perspectives on the implementation of a generic P-PROM in clinical outpatient care? (Table 2).

**Table 2:** Perspectives on the implementation of a generic P-PROM in clinical outpatient care (RQ1): Key themes, sub themes, and illustrative quotes

Theme	Sub-theme(s)	Illustrative quote(s)
Potential impact on clinical encounter	Discussing in clinical encounter	*Quote 1: “I think if there was a problem, I’d like to talk about it and if there wasn’t a problem, it would probably feel a bit like a ticking the box process.” Pt 8, Caregiver* *Quote 2: “If you’re just looking at the medical condition rather than the quality of life, you can miss out on a lot of really important information particularly for the young person… but as she gets that bit older, there’s going to be times where she’s not going to want me in the room, or I’m going to be answering questions that upset her.” Pt 18, Caregiver* *Quote 3: “I find it conceptually interesting. I think there’s some areas that we can hesitate to talk about in our work if we’re not sure how much we can do to address them, …are you opening a can of worms ….” Pt 3, Service Provider*
	Generic P-PROMs potential use in clinical encounter	*Quote 4: “If people aren’t asking them those questions [P-PROM] and they’re only asking about their tummy pain or their toileting habits or their, … fevers or vomiting history …, they [patients] maybe don’t think that stuff is meaningful to the doctors and nurses and whoever is asking these questions when it actually might be.” Pt 15, Service Provider* *Quote 5: “a lot of the medications and things have side effects that you probably don’t really measure. …we measure the obvious ones like the effect on the white blood cells or on the magnesium levels … but we don’t really say, “Oh, but does it affect your sleep or your mood,” … we might say, “maybe we’ll try a different medication that might not affect your sleep, but might work just as well… And so, I think having an understanding of what treatments can affect children, and then what that effect has on their greater self, not just their hospital self.” Pt 15, Service Provider* *Quote 6: “I think it might help them understand our situations better and with the treatments” Pt 23, Adolescent* *Quote 7: “But in relation to shared decision making between patient and clinician… I think a survey like that could be a really good starting point to help your mind start exploring the issues.” Pt 13, Caregiver* *Quote 8: “If you don’t realise that something’s trending downwards or upwards because you haven’t thought about it, like let’s say it’s every year and you don’t notice that that’s gradually getting worse or getting better, you might lean on treatment that isn’t considering the impact,” Pt 18, Caregiver* *Quote 9: “would assume that having an improved quality of life improves the likelihood that you’re going to have patients that would engage in health services that will be adherent to recommendations or medications that they’re taking.” Pt 2, Service Provider* *Quote 10: “transplant is incredible but you know, as time goes on and we realise the impact across different parts of patients’ life and I guess those sort of quality of life and those wellbeing things and how it impacts their schooling, their relationships with their friends, we could have … been able to intervene in a way that… helped that child so they perhaps didn’t have to go through that..” Pt 15, Service Provider* *Quote 11: “The hard thing about the generic tools is that it’s perhaps a little bit harder to know how I would use those data. … I know by all asthma metrics their asthma’s under really good control, but then all of a sudden, their generic quality of life gets worse, I’m not really sure what I would do with that..” Pt 17, Service Provider*
	Generic P-PROM information compared to clinical information or condition specific P-PROM information	*Quote 12: “I’m not saying this is right or wrong, but in the clinics we tend to focus really a lot on disease control, and then if you get to having to make a decision where there’s not a clear right answer from a disease control point of view, and you’re trying to weigh up two options, that’s when we often bring in, because there’s two options here, it’s like whichever one you think will give you the best quality of life, depending on what quality of life is for you guys.” Pt 17, Service Provider* *Quote 13: “Yes, generic is good but … it doesn’t capture all the outcomes that are important specifically for the cohort ….” Pt 15, Service Provider*
“Getting participants to fill them out”: Practicalities for P-PROM completion	Introduce purpose of generic P-PROM to family from trusted clinical team	*Quote 14: “I certainly wouldn’t normally say some of the things to a stranger, … but it’s for a really, really good cause, so I think it’s worth it” Pt 18, Caregiver* *Quote 15: “I know that when I was doing them, I sort of didn’t feel like they were for me, I felt like they were data collecting for, I don’t know, for whatever reason … Where it would be good if I got it first, ‘Prior to your appointment could you fill this in to help us’… then I would feel like oh wow this is something they’re interested in about [my child].” Pt 8, Caregiver* *Quote 16: “watching medical dramas on TV, ‘quality of life’ is the word they use when somebody has had a life altering illness or collision or something like that and it’s always with a negative connotation… And then when you go into the questions, they are not like that. I hope he’s got a good quality of life or else I’m not doing a very good job as a parent.” Pt 8, Caregiver* *Quote 17: “I think the main thing would be people feeling a bit self-conscious about it and worried that some of the answers might put a bad picture on them, when it wouldn’t, but they might feel a bit uncomfortable answering those.” Pt 23, Adolescent*
	Before appointment	*Quote 18: “Would it be helpful if it was sent to you, …maybe a week before your appointment? So, you had a bit of time to think about it and do it in your own space.. I know that the hospital environment there can be a lot going on.” Pt 11, Caregiver* *Quote 19: “but maybe it isn’t the most efficient way to use clinic time … If you want to get more data from them, and what’s going on… then doing that before clinic.” Pt 2, Service Provider*
	Quick and easy to complete	*Quote 20: “As long as it’s a pretty easy and quick thing to do, then I’m happy.” Pt 17, Service Provider* *Quote 21: “I think one of the main things is more of a logistic obstacle, in that it relies on people being signed up to their EMR [electronic medical record] portal.” Pt 2, Service Provider*
“When you ask, you want them to help”: Practicalities for P-PROM use	When you ask, you have to do something about it	*Quote 22: “I would want there to be suggestions or recommendations. I wouldn’t want it just to be tokenistic, I wouldn’t want it to be just filling out a form so that the hospital can understand how these issues go. I would want it to then be discussed down the track and said, “Okay, so this area’s not so good, here are some potential things that you can do for this area,” and stuff like that.” Pt 13, Caregiver* *Quote 23: “If you’re going to do it, and they flag that there’s a really low area in their quality of life, but then you don’t address that, then it becomes a bit less valuable.” Pt 2, Service Provider*
	IT infrastructure	*Quote 24: “so when we do them in clinic, they’re very easy. We just have it on EMR [electronic medical record], so we just click a button and talk through it with them.” Pt 10, Service Provider*
	Clinician buy in, education and support	*Quote 25: “A lot of lack of understanding, a lot of, … time-poor people, and I think we need a lot more education I think about the benefits of PROMs from a patient level, from a clinician level … they don’t really think about how even some of that data might be really relevant…” Pt 15, Service Provider* *Quote 26: “if we were going to implement that as a standardised thing in clinics, having some way to provide clinicians with resources for what they do with that information” Pt 2, Service Provider*
	Families receiving results	*Quote 27: “what I would like to see with that summary is, for example, if it came up that [CHILD] really struggles doing her usual activities, that then there’d be a link to, these are the places you could go to for support for that.” Pt 11, Caregiver* *Quote 28: “I think it would be really, really helpful for us and for other caregivers as well. being able to share that with them [other service providers outside hospital] as well, would be really, really helpful just so that we don’t then have to repeat all that information.” Pt 18, Caregiver*
	Patients have individual baselines and goals	*Quote 29: “Maybe a one off [PROM response] or a clinician that didn’t understand the whole picture of the patient might get alarmed with certain responses…. You’d have to take into consideration what the patient norms are before you panic people I guess with responses.” Pt 20, Service Provider*

#### Potential impact on clinical encounter

*Discussing in clinical encounter:* All participants were open to discussing use of HRQoL in clinical encounters, however, some only wanted to discuss it if there was a concern (quote 1). Several caregivers expressed the view that these discussions should be a standard (quote 2). Some service providers had considered discussing HRQoL with their patients, however, were concerned this may open a ‘can of worms’ (quote 3).

*Generic P-PROMs potential use in clinical encounter:* A wide range of potential uses were discussed, including improved: clinician-patient communication (quote 4), decision making (quote 5–7), outcomes over time (quote 8), and patient engagement (quote 9). Uses also included detecting new health problems (quote 10) and more holistic care (quote 4). Some service providers saw detecting new health problems as barriers to using generic P-PROMs, feeling this was out of their scope (quote 11).

*Generic P-PROM information compared to clinical information or condition specific P-PROM information:* There was an underlying belief from service provider participants that clinical outcomes or condition specific P-PROMs would be prioritised over generic P-PROM information (quote 12–13).

#### “Getting participants to fill them out”: practicalities for P-PROM completion

Participants had reflections on factors that might impact generic P-PROM completion.

*Introduce purpose of generic P-PROM to family from trusted clinical team:* One caregiver of a patient who had previous experience completing a P-PROM noted that trusting the care team and being clear on its use were key enablers (quote 14). Another caregiver noted that when previously completing a P-PROM, they didn’t understand the reason for completing it, which was a barrier (quote 15). Participants also noted that the term ‘quality of life’ might be a barrier to completion, as it sounds serious, and they may be concerned about judgement (quote 16–17).

*Before appointment:* Caregivers and adolescent participants noted that the hospital environment can be stressful and being given the opportunity to complete the P-PROM prior to their appointment would be an enabler (quote 18). Service providers felt that completing the P-PROM during an appointment would be a barrier to completion (quote 19).

*Quick and easy to complete:* Participants wanted the P-PROM to be short and easy to complete (quote 20). Several service providers shared experience using a patient portal system that was hard for families to sign up to and hence was a barrier to P-PROM completion (quote 21).

#### “When you ask, you want them to help”: practicalities for P-PROM use

*When you ask, you have to do something about it:* There was a strong consensus among participants that if families are being asked to complete a generic P-PROM, this information needs to be used to inform their clinical care (quote 22–23).

*IT infrastructure:* Participants noted that utilising IT infrastructure could ensure P-PROM results are easy for clinicians to see and hence use. They noted that sitting within existing systems would be an enabler (quote 24).

*Clinician buy-in, education and support:* Service providers discussed a lack of understanding about P-PROMs and a lack of buy-in from colleagues that may be a barrier (quote 25). Participants noted clinicians need education on the benefits of P-PROMs and resources to support acting on generic P-PROM results (quote 26).

*Families receiving results:* Caregiver participants noted the importance of families receiving the generic P-PROM results so they can hold and act on that information themselves or share it with other service providers (quote 27–28).

*Patients have individual baselines and goals:* Participants noted that for generic P-PROM information to be used meaningfully in the clinical encounter, it is important to consider that all patients are unique and will have different baselines and goals (quote 29).

- RQ2, What are adolescent patient, caregiver, and service provider perspectives on the EQ-5D-Y-5L as a generic P-PROM for use in clinical care? (Table 3).

**Table 3:** Perspectives on the implementation of a generic P-PROM in clinical outpatient care (RQ2): Key themes, sub themes, and illustrative quotes

Theme	Sub-theme	Illustrative quote(s)
Scoring and displaying results	Scoring	*Quote 30: “I’d want to see how they’d responded to each of them [5 P-PROM items].” Pt 3, Service Provider* *Quote 31: “even in people who seemingly have overall good quality of life, they could still be actually struggling in one area. So maybe in all people it’s really helpful to see what the breakdown is [for each P-PROM item], and where they’ve rated themselves.” Pt 2, service provider* *Quote 32: “I think it would be good to see if there’s any progress or if it’s getting worse, to track that would be good.” Pt 23, Adolescent*
	Benchmarking	*Quote 33: “I guess benchmarking broadly can give you a sense of how much to worry about things. Like it’d be good to have some broad sense if we’re introducing something like this, oh most kids say they have a little bit of pain, or not…” Pt 3, Service Provider* *Quote 34: “I would love that information. That would help me a lot. But I’m just imagining if I was her age and I’ve got a comparison to the general population, like even school reports and things like that where they get compared to the general population, are they average, are they above, are they below, can be difficult for someone who’s not in the average category.” Pt 18, Caregiver*
	Prioritisation of items	*Quote 35: “the particular interest might be one thing and next year it’s a different thing …as she grows.” Pt 18, Caregiver* *Quote 36: “I’d be worried about missing something. It’s important for us to…know the preferences but I guess overall, it’s probably important to have all that data that we have captured in case we are missing something.” Pt 18, Caregiver*
Characteristics of instrument	Scale and structure	*Quote 37: “So I guess the reason that I like it, is because it breaks it down into those categories and gives you a bit more room than some of the other questionnaires. And I also like the scale at the very end, the one to 100. I think that gives you a lot of room to consider all of the different factors.” Pt 18, caregiver* *Quote 38: “For the last 0 to 100 question [referring to EQ VAS]. I reckon it would be better to do 0 to 10. Because I think some children might find it difficult to visualise a larger number.” Pt 22, Adolescent*
	Recall period	*Quote 39:”I think for our patient cohort, ‘today’ is too narrow, because you know, conditions can change quite quickly, … I think a longer period of time would probably be more [useful] for our patient cohort…” Pt 15, Service Provider*
	Item and level wording and negative framing	*Quote 40:“I think that’s really nice without being too confronting for patients or too burdensome… I think the questions are really clear” Pt 15, Service Provider* *Quote 41: “I think people might struggle to differentiate if I have some problems or if I have a little bit of a problem. Like, that sounds like essentially the same things.” Pt 22, Adolescent* *Quote 42: “If you’ve got a child with a chronic illness who can’t do that then you are just kind of reinforcing the whole, these are all the things like he can’t do.” Pt 8, Caregiver*
Usefulness of EQ-5D-Y-5L and individual items	Relevance of overall instrument in clinical care	*Quote 43: “My first thought it’s actually a really lovely simple snapshot of what’s going on you’ve just got this really great generic tool that has just some simple but really important information in there that you can really… dive into if you need to” Pt 15, Service Provider* *Quote 44: “feel like it’s covering quite a good range of activities…. And even if the question didn’t twig and, those types of things may get you to think and say, oh look, I know it wasn’t on the form, but this is happening.” Pt 4, Caregiver* *Quote 45: “I reckon in the questionnaire, you could add an optional comments section for each multiple choice. So, if someone is not feeling very happy but it is because they are going through year 12, that is not unnormal, so I don’t think it is a cause for concern…. So, the comment would be useful for providing context, but I think it should be optional.” Pt 22, Adolescent*
	Most and least useful items	*Quote 46: “the first one walking around, that can definitely be impacted by asthma because we have a lot of patients who become breathless and they can’t participate in activities… the washing and dressing and things is probably not quite so applicable to our patients. But I mean, we’re very specific clinic. So doing usual activities, going to school, hobbies, all of that. Very, very relevant… not necessarily pain… And worried, sad or unhappy. I think there’s definitely in some of our kids an element of feeling that way.” Pt 10, Service Provider* *Quote 47: “I think that the doing the usual activities and having pain or discomfort, those are probably the big ones” Pt 18, Caregiver*
	Extra item	*Quote 48: “There’s obviously nothing specific to like having to do any medical things, as health-related quality of life stuff is, you know, how am I finding my medical treatments? Am I finding them difficult or taking my medications, are there side effects, or, you know, that kind of stuff.” Pt 15, Service Provider* *Quote 49: “Particularly the colorectal condition would be. Yeah, obviously there’s a lot of social impact with this, continence and things like that. So yeah, in an ideal world that would be a good one.” Pt 18, Caregiver* *Quote 50:“I’m thinking I guess they talk about emotions and things like that, but not necessarily like social impact. That’s something that comes up a little bit with my child’s condition. So that’s a good one to factor in as well. And that [referring to EQ VAS] just allows you to do that without specifically asking a question about it. If you ask questions about all of the things, the survey would just be endless.” Pt 18, Caregiver*

#### Scoring and displaying results

*Scoring:* Most participants did not want EQ-5D-Y-5L responses combined into a single score, preferring responses be displayed by item (quote 30) because it was more informative. Service providers reported wanting to easily see where patients were reporting problems (quote 31). All participants wanted EQ-5D-Y-5L responses displayed over time (quote 32).

*Benchmarking:* Benchmarking EQ-5D-Y-5L responses against a general population or children with the same condition was something some participants were open to, especially service providers. Service providers shared that benchmarking might help them understand how much of a concern a certain response was (quote 33). Some caregivers noted they might find benchmarking helpful, however, some shared concerns it may impact a child’s self-esteem if they were below a benchmark (quote 34).

*Prioritisation of items:* Participants shared that individual preferences would be more appropriate than group preferences, but that individual preferences would change over time which may be burdensome to capture (quote 35) One caregiver noted that if certain EQ-5D-Y-5L items were prioritised, they would be concerned the other items might be missed (quote 36).

#### Characteristics of instrument

*Scale and structure:* Participants generally liked the EQ-5D-Y-5L levels, with the adolescents finding they had room to reflect their health and caregivers finding they had room to reflect their child’s health on the levels (quote 37). One adolescent participant noted that children may find the scale of the EQ VAS hard to understand (quote 38).

*Recall period:* Some participants felt the recall period of the EQ-5D-Y-5L was too short and that a longer recall period may be more useful in their clinical context, especially for conditions that fluctuate (quote 39).

*Item and level wording and negative framing:* Most participants found the wording of the EQ-5D-Y-5L clear and easy (quote 40). However, a few participants thought the ambiguity of the level wording may be tricky for some (quote 41). There were concerns about the negative framing of the EQ-5D-Y-5L wording and how this may impact a child’s self-esteem (quote 42).

#### Usefulness of EQ-5D-Y-5L and individual items

*Relevance of overall instrument in clinical care:* Participants felt the EQ-5D-Y-5L captured a broad “snapshot” of health that could instigate further discussion (quote 43) or could prompt patients and caregivers to think more broadly about health (quote 44). Several caregiver and adolescent participants reported wanting the ability to add extra context to their responses (quote 45).

*Most and least useful items:* Participants discussed which items they would find more and less useful in the clinical context (quote 46–47). There was great variation between participants depending on the condition or individual.

*Extra item:* Common themes for what participants would like to see as an additional item included how the child’s health care/interventions/medications is impacting them (quote 48), as well as social/relationships (quote 49). Other suggestions included eating, sleeping, toileting, and schooling. One participant noted that the VAS acts as a catch all, with anything not captured by the items likely being captured by the EQ VAS (quote 50).

**Fig. 1:**
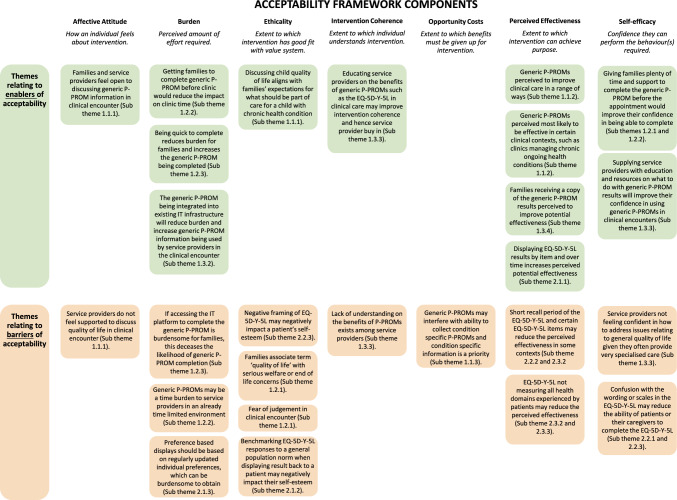
Themes mapped to the acceptability framework components. Graphic produced using Canva. Figure informed by Theoretical Framework of Acceptability (TFA) components [35]

- RQ3, How can these perspectives inform the co-design routine use of EQ-5D-Y-5L in clinical care? (Table 4 and Fig. 1).

**Table 4:** Summary of themes and sub-themes by research question

Aim	Theme	Sub-themes
RQ1, What are adolescent patient, caregiver, and service provider perspectives on the implementation of a generic P-PROM in clinical outpatient care?	1.1. Potential impact on clinical encounter	1.1.1. Discussing in clinical encounter 1.1.2. Use in clinical encounter 1.1.3. Use compared to clinical information
	1.2.“Getting participants to fill them out”: Practicalities for P-PROM completion	1.2.1. Introduce to family from trusted clinical team 1.2.2. Before appointment 1.2.3. Quick and easy to complete
	1.3. “When you ask, you want them to help”: Practicalities for P-PROM use	1.3.1. When you ask, you have to do something about it 1.3.2. IT infrastructure 1.3.3. Clinician buy in, education and support 1.3.4. Families receiving results 1.3.5. Patients have individual baselines and goals
RQ2, What are adolescent patient, caregiver, and service provider perspectives on the EQ-5D-Y-5L as a generic P-PROM for use in clinical care?	2.1.Scoring and displaying results	2.1.1. Scoring 2.1.2. Benchmarking 2.1.3. Prioritisation of items
	2.2. Characteristics of instrument	2.2.1. Scale and structure 2.2.2. Recall period 2.2.3. Item and level wording and negative framing
	2.3. Usefulness of EQ-5D-Y-5L and individual items	2.3.1. Relevance of overall instrument in clinical care 2.3.2. Most and least useful items 2.3.3. Extra item

Themes are summarised in Table 4. Participants were generally supportive of using generic P-PROM information in clinical discussions, with participants identifying several potential benefits (Theme 1.1). It was felt that generic P-PROMs shouldn’t be implemented without supports for families to complete them (Theme 1.2), and a genuine effort to use such information in the individual patient-clinician encounter (Theme 1.3). Service providers felt ill-equipped to use generic P-PROMs without dedicated resources and education (Theme 1.3). Participants were largely supportive of using the EQ-5D-Y-5L as a generic P-PROM in clinical care, with participants preferring responses be displayed by item (Theme 2.1.1). Although some participants were concerned about the short recall and negative framing of the EQ-5D-Y-5L, most valued it being short and easy to complete (Theme 2.2). All participants felt positively that the EQ-5D-Y-5L captured a snapshot of health, with participants able to describe how different items may be relevant in their clinical context (Theme 2.3).

Figure 1 displays enablers and barriers to the potential acceptability of using the EQ-5D-Y-5L, in clinical outpatient care by mapping identified themes summarised in Table 4 to the seven components of the Theoretical Framework for Acceptability: 1) Affective Attitude, 2) Burden, 3) Ethicality, 4) Intervention Coherence, 5) Opportunity Costs, 6) Perceived Effectiveness, and 7) Self-efficacy [35]. Mapping themes as potential enablers and barriers of acceptability helps inform future co-design stages by ensuring future design discussions address key aspects of acceptability. For example, Fig. 1 outlines that the generally supportive attitude of participants regarding the use of generic P-PROMs in clinical discussions (Sub-theme 1.1.1) was an enabler of stakeholder attitudes to intervention acceptability, whilst service providers feeling a lack of support to discuss general quality of life with patients (Sub-theme 1.1.1) was a barrier. Hence a key focus of future co-design stages should focus on how the intervention can be designed to overcome this barrier and ensure service providers do feel supported to meaningfully discuss quality of life issues in clinical encounters.

## Discussion

This study is the first to describe adolescent patient, caregiver and service provider perspectives on the implementation of a generic P-PROM, specifically the EQ-5D-Y-5L. Participants shared perspectives on the importance of integrating with existing IT systems, having trusted providers introduce P-PROMs to families, giving families the opportunity to complete P-PROMs before appointments, and ensuring resources are available to support the use of P-PROMs in clinical care. Results demonstrated that clinicians and consumers are open to discussing HRQoL in patient-clinician encounters and can identify how this could be useful. Both clinicians and consumers shared perspectives on the use of the EQ-5D-Y-5L in patient-clinician encounters that reflect the common benefits (applicable to wide range of children, short, easy to complete) and pitfalls of a generic PROMs (not all aspects of health captured, broad, lack of clarity on how to address in specialist clinical context) [7, 20].

In a recent systematic review of P-PROMs in clinical care, no studies had included the patient and caregiver voice in the design of the clinical P-PROM program [12], which may limit the success of such a program [17]. Previous studies that have involved stakeholders only included service providers or caregivers [36–38]. Furthermore, no study has explored views of using the EQ-5D-Y-5L in paediatric clinical care. However, the adult version of the instrument, the EQ-5D-5L, has been qualitatively explored with Australian service providers in the adult care sector [20]. The findings are somewhat comparable to this study, with both finding similar barriers (i.e. highly generic items, short recall period) and enablers (quick and easy to complete, aligns with expectations of care, and measuring relevant aspects of health) to acceptability [20]. However, this study also identified additional barriers (service providers not having the education or resources to discuss generic P-PROM results with patients, patient/caregiver fear of judgement from service providers, negative framing of EQ-5D-Y-5L, lack of clarity about EQ-5D-Y-5L levels, desire to provide additional context) and enablers (integration with IT systems, completion before appointment, families receiving a copy of EQ-5D-Y-5L results, supports/education for families, and supports/education for clinicians) of acceptability. These variations likely reflect the different clinical contexts (adult versus paediatric) and participants (clinician only versus clinicians and consumers) in the two studies, highlighting the importance of including the consumer perspective [20]. Some of these additional factors, such as supports/education for patients, supports/education for clinicians, integration with clinical workflow, and a lack of resources to use P-PROM information in clinical care, were however captured in two recent studies [18, 19]. This highlights that some factors are shared across contexts, whilst others are specific to the local context.

The study has several limitations. Firstly, parents were able to be present during adolescent interviews to ensure adolescents felt comfortable. However, during one interview, the parent did most of the talking, likely limiting the contribution of the adolescent [39]. Secondly, the voice of younger children, fathers, and of those who speak a language other than English was not captured. Thirdly, participants from clinics planning to be involved in latter design work were prioritised (colorectal surgery, asthma, sleep, and encopresis clinics). Consequently, results may not be transferable beyond these clinics. Fourthly, only three adolescent patients took part, which may be a limitation of this study, further research may benefit from a larger sample of adolescent patients. Finally, there is evidence to suggest that involving stakeholders in the choice of a P-PROM may be beneficial [16, 17]. However, as this study was limited in scope, the EQ-5D-Y-5L was presented to stakeholders in this qualitative study to determine if it was appropriate for use in clinical care, rather than providing stakeholders with a range of P-PROMs to choose from. A strength of the participants recruited as part of this study is that they are from a wide range of clinical settings, including medical and surgical, representing chronic ongoing and episodic conditions. Another strength of this study is the use of rigorous qualitative methods, including use of frameworks [35].

Participants in this study indicated that displaying EQ-5D-Y-5L responses by item would be preferred to summarising into a single score. Similar scoring and display approaches have been used in routine adult care in Canada [31], and in paediatric care in the Netherlands [40]. This approach differs to how they are commonly scored in research and health technology assessment contexts, where responses are summarised into a single score using preference weights derived from the general population [15]. As previous research has largely focussed on preference weighted scoring approaches, there is a lack of research on scoring and display approaches for clinical care. Consequently, a key focus of future design phases needs to be on how score and display EQ-5D-Y-5L meaningfully in clinical paediatric care. By mapping the themes identified in this study into potential barriers and enablers of acceptability, future co-design stages can focus in designing a scoring and display system that maximises enablers (displaying results by item, families and clinician open to more holistic care approach, providing results to family as well as clinician) and minimises barriers (negative framing of EQ-5D-Y-5L, stigma regarding the term ‘quality of life’, benchmarking to general population causing potential self-esteem issues). Further modifications should also be considered to increase EQ-5D-Y-5L’s acceptability in clinical contexts. This could include adding bolt-on questions to capture other areas of concern, the ability for patients to provide additional context or emphasis, and ways of addressing the negative framing of the instrument.

## Conclusions

The findings from this study highlight that clinicians and consumers are optimistic about the potential impact of using a generic P-PROM like the EQ-5D-Y-5L in routine clinical care, suggesting that advancing to further implementation design phases is warranted. These findings also highlight key areas of focus for future design. Participants were clear that if we ask families to engage with a generic P-PROM like the EQ-5D-Y-5L, it is essential this information is used meaningfully in clinical encounters. Additionally, participants highlighted that families need to feel supported, and clinicians need to feel confident in using the information in clinical care. Finally, participants indicated that displaying EQ-5D-Y-5L responses by item would be preferred to summarising into a single score. Future design work should focus on how the score and display the EQ-5D-Y-5L meaningfully for use in clinical care. This co-design work will be progressed in Phase 2 this P-PROM ROCK project. Once designed, prototypes should be trialled to assess feasibility, acceptability, and impact on clinical care.

## Supplementary Information

Below is the link to the electronic supplementary material.

Supplementary file1 (PDF 202 KB)

Supplementary file2 (PDF 418 KB)
