# Supplementary material for: Use of a generic Paediatric Patient Reported Outcome Measure (P-PROM) in Routine hospital Outpatient Care for Kids (ROCK): A qualitative exploration of adolescent, caregiver and service provider perspectives (P-PROM ROCK Phase 1)
Source: Qual Life Res. 2025 May 14;34(8):2189–204. doi: 10.1007/s11136-025-03990-3 (PMC12274235; doi:10.1007/s11136-025-03990-3)
Supplement: Supplementary file 1 — Supplementary file1 (PDF 202 KB) [file 11136_2025_3990_MOESM1_ESM.pdf]

Supplementary File 1. Semi-structured Interview Guides

Contents

1. Service Provider - Semi-Structured Interview Guide 1
2. Adolescent - Semi-Structured Interview Guide 5
3. Caregiver - Semi-Structured Interview Guide 8

1. Service Provider - Semi-Structured Interview Guide

Topic	Checklist – ask if applicable
**Introductory questions regarding awareness of P-PROMs and providing definitions.**	Today we will be talking about Paediatric Patient Reported Outcome Measures (or P-PROMs), these are also often referred to as health related quality of life questionnaires. Have you heard or thought much about these before?<br><br>[After discussion]<br>When I talk about P-PROMs, I am referring to is a set of questions that aims to capture a child's health and wellbeing from their perspective that has been widely used and tested. Where the child is young, there may be an option for a parent to report on the set of questions, however, the aim is still to understand the child's health and wellbeing.<br><br>When I refer to "generic P-PROMs" I mean a set of questions designed to understand a child's perspective of their **overall** health and wellbeing, these are often called generic health related quality of life questionnaires. These generic P-PROMs ask questions about health that are common to most children. For example, the PedsQL generic module is an example of a generic P-PROM.<br><br>When I refer to "condition specific P-PROMs" I mean a set of questions designed to understand a child's perspective of their particular condition from their perspective. These condition specific P-PROMs ask questions specific to that particular condition. For example, the PedsQL asthma module is an example of a condition specific P-PROM.<br><br>[Only if needed will I say the below]<br>There is also a very similar term called 'PREMs', which refers to patient reported experience measures. These differ to PROMs because they aim to capture a patients experience with a health service whereas as PROM aims to capture the patient's perception of

	the health and wellbeing. We are really interested in hearing about your views on PROMs.
Experience using P-PROMs	Do you have any experience using P-PROMs? This could be in a research project or as part of clinical care.<br><br>[If yes, use below prompts if applicable]<br>Can you tell me a bit more about the type of P-PROM and the setting?<br>• What type of setting was it?<br>• What type of P-PROM was it?<br>• Do you remember the name of the P-PROM?<br>• When was this?<br>• How did you decide on this P-PROM?<br><br>Can you tell me a bit about how you used the P-PROM information?<br>• What was your objective for using the P-PROM?<br>• How did you use the P-PROM information for your objective?<br>• Can you think of any other ways the information could have been used?<br><br>Thinking about your objective for using the P-PROM, was the P-PROM information useful?<br>• How was it useful to you? Can you provide examples?<br>• Can you think of any other ways the information could have been useful?<br><br>Were there any challenges for you when collecting, using, and reporting back the P-PROM information? *People have already talked about challenges with EMR integration, so it would be great to hear about other challenges.*
Views on P-PROMs in clinical care and how could they be used.	Do you talk about quality of life with your patients?<br><br>Do you think generic P-PROMs (or quality of life questionnaires) would be useful in the clinical care you provide?<br>• Why or why not?<br>• How might quality of life information be useful to you or your patients? This could be at the individual patient level or the aggregate level.

	If you lived in a world where anything was possible in terms of budget and feasibility, what are all of the ways you think of that <u>generic P-PROMs</u> could be useful in clinical care?<br>• We have done a review of the literature and discussed potential uses with other people and we have a bit of a list I would like to share with you. [Share screen with list]. Have you thought these potential uses? [Referring to screen shared with list].<br>• Which of these would be the most important to you and why?<br><br>Can you imagine any ways you might use this information in any hospital reporting or accreditation?
Views on EQ-5D-Y as P-PROM for use in routine clinical care.	One example of a generic P-PROM is the EQ-5D-Y, I have shared this on the screen so you can see what it looks like. I have received funding for my PhD to focus on this specific P-PROM. Would it be ok if I get your views on the EQ-5D-Y specifically now? I am interested in hearing any additional thoughts you have on this that we may not have talked about yet or that may be different to what we have already talked about.<br><br>Before I ask for your thoughts on this P-PROM, I wanted to mention that we have put a lot of work into checking this P-PROM performs well. I have shared screen with a summary of the results from this work and I can leave these results with you.<br><br>Looking at the EQ-5D-Y, what do you think of this generic P-PROM?<br>• Are there any items on here you think you would already have information on?<br>• Which items do you not ask about or have information on for your patients?<br>• Do you think these items capture things important to your patients?<br>• Which of these items do you think would be most <u>useful</u> to you in your clinical care?<br>• Do you think there is anything missing from this P-PROM that would be important to your patients?<br>• If you could add any generic items (i.e., not condition specific) to this P-PROM, what would it be and why?<br><br>Looking at the EQ-5D-Y, what do you think about this generic P-PROM being used in the clinical care you provide?<br>• Would it be useful? Why or why not?<br>• What do you think you would do with this information in your clinical encounters?

<table>
<tr><td></td><td>
We talked before about all the potential ways generic P-PROMs could be used in clinical care. [Share screen with list]. Are there any ways you think wouldn’t be possible with this P-PROM? Or are there any you now think would be possible? Could you tell me more about this.
Looking at the EQ-5D-Y, how would you want to see and interpret this information when seeing individual patients? [Use prompts below if needed]

Are you most interested in the total score or the results by items? Can you tell me why that is?
How would you interpret this information?
Are you interested in seeing results compared to other children? If so, what other children and why? (i.e., population norm, similar condition, similar diagnosis stage, similar treatment stage, similar age)
Are you interested in seeing results over time? If so, what time intervals would be important to you and why? I.e., Aging, treatment, exacerbations
If we were just to provide one standard report, what is the most important information to have in that report and why?
When do you want to see this information? Why is it important for you to see it then?
How frequently would you like this P-PROM collected?
What intervals would be important to you? i.e., each appointment, exacerbations, time intervals, pre and post treatment. Why are these intervals important to you?

What evidence about this P-PROM would you want to see for you to use this in your practice?
</td></tr>
<tr><td>Barriers or enablers for using generic P-PROMs in clinical care.</td><td>
Can you think of any barriers, outside of integrating with the EMR, for using generic P-PROMs in clinical care?
For example, some people have mentioned the time for clinicians to review and discuss these with patients as a barrier, as well as not knowing what to do with this information once they have it, the cost of using P-PROMs and not having the capacity or resources to analyse and report on the aggregate data.
Can you think of any enablers to overcome these barriers?
What would make you more likely to use generic P-PROMs in the clinical care you provide?
</td></tr>
<tr><td>Final thoughts?</td><td>Is there anything else you wanted to mention that we may not have already discussed?</td></tr>
</table>

2. Adolescent - Semi-Structured Interview Guide

Topic	Checklist – ask if applicable
Introductory questions and providing definitions.	Today we will be talking about quality of life questionnaires.<br><br>Have you heard of quality of life or quality of life questionnaires before?<br><br>[After discussion]<br>There are a lot of ways we can measure health, sometimes we use things like a blood tests. We can also use things like quality of life questionnaires. We think quality of life questionnaires are good because they can tell us what a child or adolescent thinks about their own health.<br><br>When I talk about quality of life questionnaires, I am talking about a set of questions that asks a child or adolescent to tell us what they think about their overall health and wellbeing. These questionnaires ask about aspects of health that are common to most children or adolescents.<br><br>I have shared my screen with an example of what a quality of life questionnaire looks like.<br><br>Do you have any questions for me about what a quality of life questionnaire is?
Experience with questionnaires.	Have you ever filled out a questionnaire as part of a visit to the doctor? This could mean filling it out before the visit or during the visit.<br>• Can you tell me a bit about this?<br>• How did you find filling out this questionnaire?<br>• Do you think filling out the questionnaire was useful to you or the doctor?
Discussing quality of life with doctor.	How would you feel about discussing overall health and wellbeing with a nurse or doctor, including emotional wellbeing and how your health impacts your daily life?<br><br>What sort of things do you want the nurse or doctor to talk with you about? For example, do you only want them to talk about your specific condition or do you want them to talk about other parts of health and wellbeing?

	How would you feel about bringing this up to your nurse doctor?<br><br>How would you feel if your nurse or doctor talked to you about this?
Views on usefulness of quality of life questionnaires.	**Do you think filling out a quality of life questionnaire before you visit the hospital clinic would be helpful? Why or why not? [Bring up list from literature if helps guide them]**<br><br>Would you want to talk about the results of the quality of life questionnaire with your nurse or doctor during your hospital clinic visit? Why or why not?<br><br>Would you want your parents to see the results of the quality of life questionnaire? Why or why not?<br><br>How do you think filling out a quality of life questionnaire before you visit the hospital clinic would be helpful to you or your doctor?<br><br>Would you rather fill out a questionnaire most specific to your condition?
Views on EQ-5D-Y.	**One example of a quality of life questionnaire is the EQ-5D-Y, I have shared this on the screen so you can see what it looks like. I have received funding for my PhD to focus on this specific quality of life questionnaire. Would it be ok if I get your views on this quality of life questionnaire specifically now? I am interested in hearing any additional thoughts you have on this specific quality of life questionnaire.**<br><br>**Looking at the quality of life questionnaire on the screen, what do you think about this?**<br><br>Do you think these questions capture things important to you and your health and wellbeing?<br><br>Which of these questions are most important to you when you think about your health and wellbeing? Why is that important to you?<br><br>Is there anything missing from this questionnaire? If it could include other questions, what would these be?<br><br>Do you think filling this out would be useful to you or your doctor when you visit the hospital clinic?<br>• Why or why not?

	• How do you think it could be useful?<br>• We talked before about all the potential ways quality of life questionnaires could be used in clinical care. [Share screen with list]. Are there any ways you think wouldn't be possible with this quality of life questionnaire? Or are there any you now think would be possible? Could you tell me more about this.<br>• Which use would be most important to you and why?<br><br>Would you want to see the results of this questionnaire?<br><br>What kind of information or results regarding this questionnaire would be most useful to you?<br><br>How would you want to see this information? For example, do you want to see:<br>• These results over time<br>• How these results compare to other children with the same condition<br>• How these results compare to a general population of children
Challenges in implementation.	We are thinking of asking adolescents and young people to fill these out when they come into the hospital clinics, can you think of any challenges we might have when we as adolescents and young people to do this?<br><br>Can you think of anything that might help us overcome these challenges?
Final thoughts.	Is there anything else you wanted to mention that we may not have already discussed?

3. Caregiver - Semi-Structured Interview Guide

Topic	Checklist – ask if applicable
Introductory questions and providing definitions.	Today we will be talking about quality of life questionnaires.<br><br>Is that something you've heard of before?<br><br>[After discussion]<br>There are a lot of ways we can measure health, sometimes we use things like a blood tests. We can also use things like quality of life questionnaires. We think quality of life questionnaires are good because they can tell us what a child or young person thinks about their own health.<br><br>When I talk about quality of life questionnaires, I am talking about a set of questions that asks a child or adolescent to tell us what they think about their overall health and wellbeing. These questionnaires ask about aspects of health that are common to most children or adolescents.<br><br>I have shared my screen with an example of what a quality of life questionnaire looks like.<br><br>Do you have any questions about what a quality of life questionnaire is?
Experience with questionnaires.	Have you ever filled out a questionnaire for your child as part of a visit to the doctor? This could mean filling it out before the visit or during the visit.<br>• Can you tell me a bit about this?<br>• How did you find filling out this questionnaire?<br>• Do you think filling out the questionnaire was useful to you or the doctor?
Discussing quality of life with doctor or nurse at hospital clinic visit.	How would you feel about discussing your child's overall health and wellbeing with the nurse or doctor at the hospital clinic, including emotional wellbeing and how your child's health impacts their daily life?<br><br>How would you feel about bringing this up to the doctor or nurse?<br><br>How would you feel if your doctor or nurse talked to you about this?<br><br>What sort of things do you want the nurse or doctor at the hospital clinic to talk with you and your child about? For

	example, do you only want them to talk about your specific condition or do you want them to talk about other parts of health and wellbeing?
Views on usefulness of quality of life questionnaires.	Do you think filling out a quality of life questionnaire before you visit the hospital clinic would be helpful? Why or why not? [Bring up list from literature if helps guide them]<br><br>Would you want to talk about the results of the quality of life questionnaire with the doctor? Why or why not?<br><br>How do you think filling out a quality of life questionnaire before you visit the doctor for your child would be helpful to you, your child or the doctor?
Views on EQ-5D-Y.	One example of a quality of life questionnaire is the EQ-5D-Y, I have shared this on the screen so you can see what it looks like. I have received funding for my PhD to focus on this specific quality of life questionnaire. Would it be ok if I get your views on this quality of life questionnaire specifically now? I am interested in hearing any additional thoughts you have on this specific quality of life questionnaire.<br><br>Looking at the quality of life questionnaire on the screen, what do you think about this?<br><br>Do you think these questions capture things important to your child's health and wellbeing?<br><br>Which of these questions are most important to you when you think about your child's health and wellbeing?<br><br>Is there anything missing in this questionnaire? If it could include other questions, what would these be?<br><br>Do you think filling this out would be useful to you, your child or your doctor?<br>• Why or why not?<br>• How do you think it could be useful?<br>• We talked before about all the potential ways quality of life questionnaires could be used in clinical care. [Share screen with list]. Are there any ways you think wouldn't be possible with this quality of life questionnaire? Or are there any you now think would be possible? Could you tell me more about this.

	• Which use would be most important to you and why?<br><br>Would you want to see the results of this questionnaire?<br><br>What kind of information or results regarding this questionnaire would be most useful to you?<br><br>How would you want to see this information? For example do you want to see:<br>• These results over time for your child<br>• How these results compare to other children with the same condition<br>• How these results compare to a general population of children
Challenges in implementation.	We are thinking of asking children, young people or their caregivers to fill these out before they attend the hospital clinic, can you think of any challenges we might have when we ask people to do this?<br><br>Can you think of anything that might help us overcome these challenges?
Final thoughts.	Is there anything else you wanted to mention that we may not have already discussed?
